# Corticosteroid Therapy in Early Postoperative Small Bowel Anastomotic Strictures: A Case Report and Literature Review

**DOI:** 10.7759/cureus.96573

**Published:** 2025-11-11

**Authors:** Mohamed A Salman, Subash Rai, Hassan Nauyan, Mohamed Tourky, Lok B Kathayat

**Affiliations:** 1 Surgery, Kasralainy School of Medicine, Cairo, EGY; 2 General and Bariatric Surgery, University Hospital Ayr, Ayr, GBR; 3 General Surgery, James Paget University Hospitals NHS Foundation Trust, Great Yarmouth, GBR; 4 Surgery, North Lincolnshire and Goole NHS Foundation Trust, Scunthorpe, GBR; 5 General Surgery, Great Western Hospitals NHS Foundation Trust, Swindon, GBR

**Keywords:** anastomotic stricture, corticosteroid therapy, femoral hernia, intestinal obstruction, intravenous hydrocortisone, postoperative complication, small-bowel obstruction

## Abstract

For early anastomotic strictures, conservative approaches involve parenteral nutrition and bowel rest, with most obstructions relieving spontaneously. Conservative approaches involve parenteral nutrition and bowel rest, with most obstructions relieving spontaneously. However, reoperation may be required for refractory cases. Corticosteroids may be effective in reducing inflammatory edema and restoring luminal patency. We report a case of a 50-year-old woman who developed small bowel obstruction following laparoscopic small bowel resection and anastomosis due to a strangulated femoral hernia. CT with oral and intravenous contrast suggested peri-anastomotic edema without evidence of leak or peritonitis. Conservative interventions in the form of nil per mouth, intravenous fluids, and nasogastric (NG) tube failed, and intravenous hydrocortisone, tapered over five days, was administered. This resulted in rapid improvement of bowel function without recurrence and avoidance of reoperation. This case highlights the potential role of short-term corticosteroid therapy as an adjunct to other conservative measures in carefully selected patients, particularly in the setting of partial obstruction without evidence of leak, to avoid the need for resurgery.

## Introduction

Early postoperative small bowel obstruction (EPSBO) is a rare but significant complication following abdominal and pelvic surgery, occurring within the first 30 days after the procedure. EPSBO is hardly distinguished from postoperative ileus, wherein a physiological bowel function may take several days to recover postoperatively [1]. While adhesions account for a considerable proportion of obstructions, common causes of EPSBO include localized anastomotic edema, kinking, or technical issues such as internal herniation or anastomotic stricture [1]. Distinguishing EPSBO from these technical complications is crucial, as management strategies differ.

The first-line therapy for these strictures involves nasogastric (NG) decompression, fluid resuscitation, and parenteral nutrition [2,3]. Nevertheless, reoperation may be required in the instance of prolonged obstruction or clinical deterioration to avoid subsequent risks of morbidity and mortality [1,4]. Due to the anti-inflammatory and anti-edematous effects of corticosteroids, they have been explored as an adjunct to conservative treatment. 

Corticosteroids have been associated with clinical improvement in encapsulating peritoneal sclerosis and malignant bowel obstruction [5], and they can reduce peri-anastomotic edema and restore luminal patency [6]. However, cases requiring prolonged administration of systemic corticosteroids might be subject to infections and impaired wound healing [7]. This underscores the effect of long-term steroids, which are known to increase leak rates and suppress immunity, while nonsteroidal anti-inflammatory drugs (NSAIDs) can induce anastomotic ulcers. 

Corticosteroids are generally not recommended as first-line therapy following bowel anastomosis because of their potential to impair collagen synthesis and wound healing, thereby increasing the theoretical risk of anastomotic dehiscence. Nevertheless, in selected cases where imaging suggests inflammatory or edematous narrowing rather than a true mechanical obstruction, short-term corticosteroid therapy may help reduce mural inflammation and improve luminal patency.

Although corticosteroids have been explored in malignant bowel obstruction and encapsulating peritoneal sclerosis, evidence regarding their role in early postoperative small bowel anastomotic strictures remains scarce. The current study demonstrated the successful use of corticosteroids in a patient with EPSBO due to suspected anastomotic edema causing anastomotic narrowing, highlighting their potential role as a safe and effective option in selected cases.

## Case presentation

**Figure 1:**
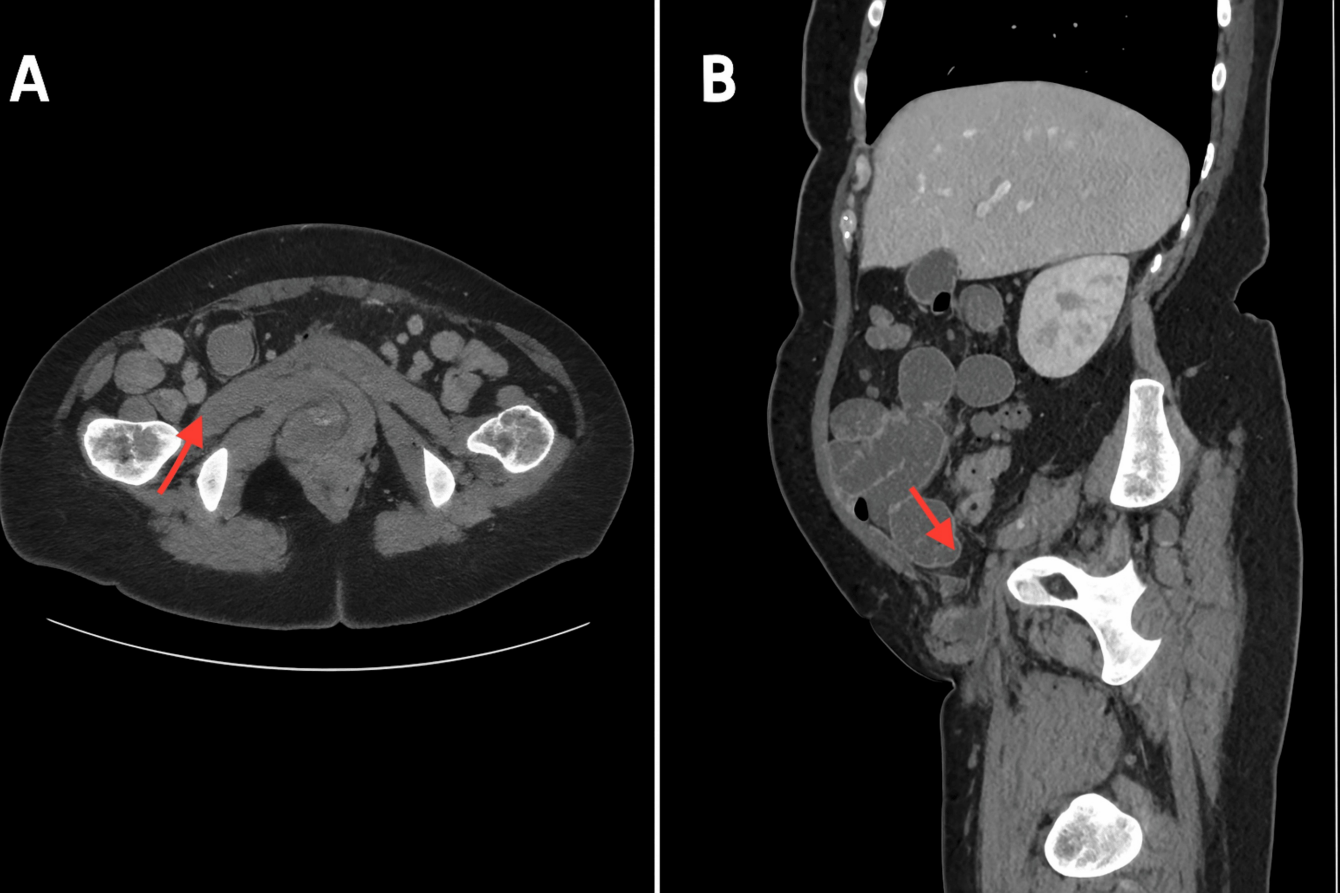
Results of abdominal CT investigation showing evidence of obstruction at the right femoral ring on the coronal (A) and sagittal (B) views Abdominal CT at presentation (coronal view A and sagittal view B) showing evidence of small bowel obstruction at the right femoral canal (arrow), consistent with a strangulated femoral hernia

A 50-year-old female patient presented to the emergency department complaining of sudden-onset abdominal pain and vomiting. On examination, she had a distended, soft abdomen and tachycardia. She had a tender, irreducible 3 * 2 cm lump on the right groin. A scar of a previous open inguinal hernia repair was noted on the left groin. The patient had a surgical history of an open right inguinal hernia repair with mesh. Laboratory workup showed leukocytosis (white blood cell count, 13 × 109/L) and an elevated C-reactive protein level (76 mg/L). Abdominal computed tomography (CT) demonstrated a high-grade obstruction in the small bowel with a transition point at the right femoral hernia, suggesting incarceration or strangulation (Figure 1).

Emergency laparoscopy showed approximately 10 cm of strangulated small bowel within a femoral hernia sac, 2 meters proximal to the ileocecal valve. The bowel was reduced by a combination of external and laparoscopic reduction of the small bowel. The reduction was challenging, and the bowel was finally retrieved from the abdomen and found to be non-viable. There was a laparoscopic incidental finding of recurrent left inguinal hernia, which was not felt clinically. A laparoscopic resection of non-viable bowel was done. The continuity was restored with a laparoscopic 45 mm stapled side-to-side small bowel anastomosis. The enterotomy was closed with 3/0 PDS in two continuous layers. We opted not to use mesh for the repair of the bilateral hernias because of the concomitant bowel resection and the risk of contamination. Instead, intracorporeal suturing was performed using 2/0 barbed sutures to close the right femoral ring and the left internal ring, addressing the incidental left indirect hernia identified during the same procedure. As this did not constitute a formal mesh hernia repair, we considered it appropriate to proceed as part of the overall operation. The mesenteric defect was closed, and a 20 Fr Robinson pelvic drain was inserted.

**Figure 2:**
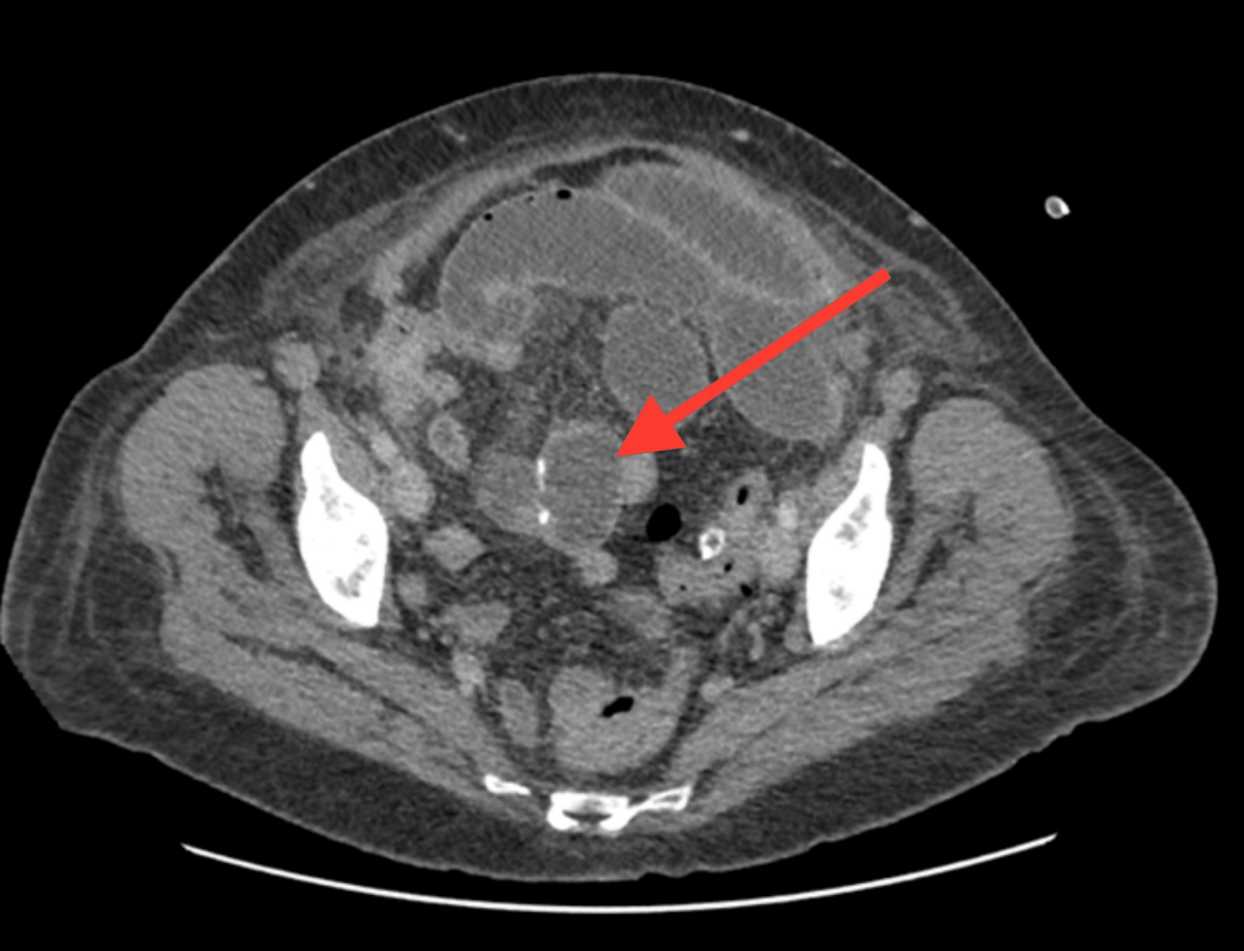
CT findings day 3 postoperative: dilated proximal loops, collapsed distal bowel, query transition point at the anastomotic site CT scan on postoperative day 3 showing markedly dilated proximal small bowel loops with collapsed distal loops. A possible transition point can be seen at the site of the small bowel anastomosis (suggesting anastomotic stricture)

**Figure 3:**
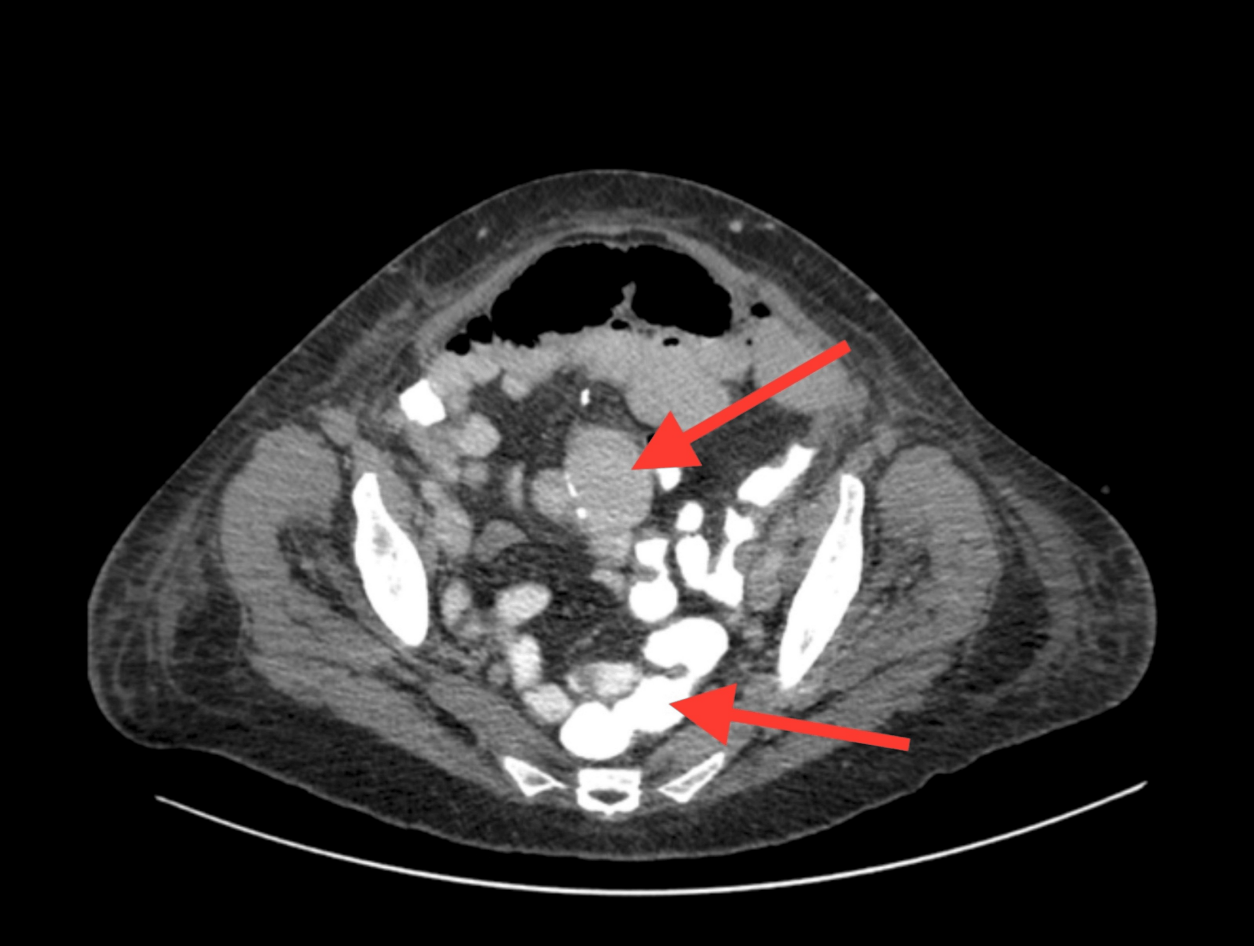
Abdominal CT with gastrografin day 6: Findings indicated persistent proximal dilatation, some gastrografin passed distally in the collapsed bowel, no signs of leak or collection Abdominal CT with oral contrast (gastrografin) on postoperative day 6 demonstrating persistent proximal small bowel dilatation. A small amount of contrast (bright fluid) has passed distally beyond the anastomosis into the collapsed bowel, and there are no signs of an anastomotic leak or intra-abdominal collection

Postoperatively, no signs of bowel recovery were apparent by day 3 (Figure 2), and the patient developed persistent abdominal distension. Other possible causes of postoperative ileus or pseudo-obstruction were carefully excluded. The patient’s serum electrolytes, including sodium, potassium, calcium, and magnesium, were within normal limits. A detailed medication review showed no recent use of opioids, anticholinergics, or other agents known to impair intestinal motility. There was no evidence of metabolic or infectious causes contributing to bowel dysmotility. These findings supported a diagnosis of EPSBO secondary to localized anastomotic edema rather than a functional ileus. Abdominal CT showed dilatation of the proximal small bowel loops with distal collapse. Such evidence raised concern for an anastomotic stricture. Conservative management with NG decompression and total parenteral nutrition (TPN) was started. However, persistent dilatation was still evident on day 6 without leak, collection, or peritonitis (Figure 3).

In view of the peri-anastomotic edema as per the radiological findings, along with the absence of systemic sepsis, a short course of corticosteroids was initiated. The regimen consisted of 100 mg intravenous hydrocortisone twice daily for three days, followed by 100 mg once daily for two days. The patient showed rapid improvement. The steroid regimen was started on day 8 postoperatively, as CT suspected anastomotic edema.

Although water-soluble contrast agents such as gastrografin can have both diagnostic and therapeutic effects in adhesive small bowel obstruction by reducing wall edema and stimulating peristalsis, we believe its role in this case was limited. The gastrografin study was performed primarily for diagnostic assessment on postoperative day 6, and no immediate improvement was observed thereafter. The patient’s clinical and radiological improvement occurred only after the initiation of corticosteroid therapy, namely, on day 9 postoperatively, suggesting that the resolution was more likely related to the anti-inflammatory effect on peri-anastomotic edema rather than to the osmotic action of the contrast agent. Nevertheless, the potential contribution of gastrografin cannot be completely excluded and remains acknowledged as a possible confounding factor.

**Figure 4:**
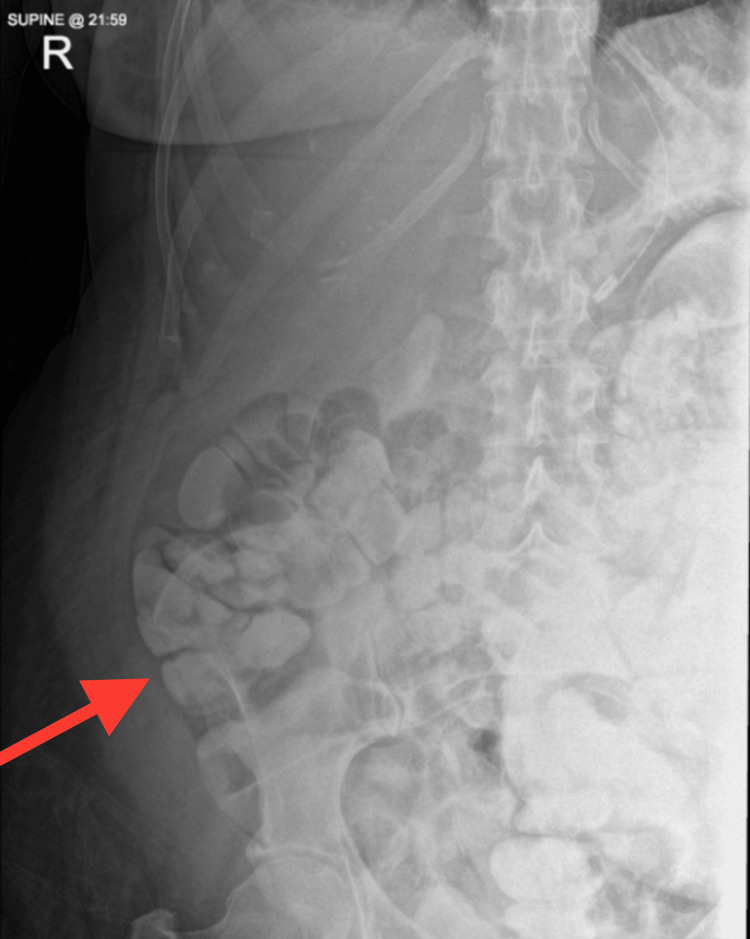
Clinical resolution was apparent on abdominal X-ray by day 8 after surgery Abdominal radiograph on postoperative day 8 showing resolution of the small bowel obstruction. The previously distended loops have decompressed, indicating restoration of bowel continuity and function after corticosteroid therapy

On day 9, normal bowel function was retained and oral intake resumed (Figure 4). TPN was discontinued on day 11. The patient was discharged 13 days after surgery, tolerating a full diet. She remained well at the three-month outpatient follow-up, tolerating a normal diet.

## Discussion

The current case report showed that a short course of systemic corticosteroids successfully managed early EPSBO caused by peri-anastomotic edema, and corticosteroid administration allowed effective recovery without the need for reoperation. Similar findings were indicated in the literature (Table 1), where case-based evidence highlighted the beneficial role of both systemic and local steroids in relieving anastomotic obstruction related to edema or stricture formation. These effects are primarily evident after the conventional measures have failed.

**Table 1:** A summary of findings for studies assessing the role of corticosteroids in early postoperative small bowel anastomosis

Study	Study type	Clinical context	Steroid intervention	Outcomes
Atie et al., 2016^[3]^	Case series (3 cases)	Postoperative edema causing obstruction (fundoplication, gastrojejunostomy, small bowel entero-enterostomy)	IV dexamethasone 8 mg/day for 72 h	Rapid resolution of obstruction, avoided reoperation in all cases
Arima et al., 2020^[8]^	Case report	Edematous anastomotic stenosis after laparoscopic gastrectomy with delta-shaped anastomosis	IV prednisolone 40 mg/day for 4 days, 20 mg/day for the next 3 days, 10 mg/day for the subsequent 8 days. Thereafter, oral steroids 5 to 10 mg/day	Improvement of stenosis, no recurrence, avoided reoperation
Toledo-Pereya et al., 1977^[9]^	Case series (10 patients	Postoperative bowel anastomotic dysfunction due to anastomotic edema (small and large bowel resections)	IV dexamethasone 0.2-0.4 mg/kg/day, given q8h for 48 h	Rapid resolution of adynamic ileus/partial obstruction within 8-24 h, no short- or long-term complications, avoided reoperation
Satomi and Sakakibara 2018^[10]^	Case series (4 cases)	Anastomotic strictures after colorectal surgery (transverse colectomy, sigmoid colectomy, colostomy closures)	IV methylprednisolone infusion (2 days) or systemic steroid monotherapy	Symptoms improved within 1-2 days in all patients, demonstrating rapid and effective resolution of strictures

For example, Atie et al. [3] reported a series of three patients with intestinal obstruction due to postoperative edema that did not respond to conservative management. The obstruction was confirmed radiologically, and all the patients were successfully managed by intravenous dexamethasone at 8 mg for three days, with complete resolution of symptoms, return of bowel function, and avoidance of reoperation [3]. Arima et al. [8] demonstrated a case with anastomotic stenosis following distal gastrectomy as revealed by contrast-enhanced CT scans. The patient was refractory to endoscopic balloon dilation and local steroid injection. Systemic prednisolone was given, and the symptoms started to improve [8]. An early study carried out by Toledo-Pereya et al. [9] assessed the effects of short courses of intravenous corticosteroids on postoperative anastomotic dysfunction due to intestinal edema after bowel resection. Systemic dexamethasone injections were given 5-10 days after surgeries, with symptomatic improvement occurring within eight to 24 hours after treatment initiation [9]. Satomi and Sakakibara [10] have also reported that short-course systemic steroids had an effective role in symptomatic resolution and recovery among four patients with postoperative colorectal anastomotic strictures. A summary of similar case-based studies is demonstrated in Table 1.

The impact of corticosteroids was apparent in reducing obstruction, even for cases with no bowel anastomotic strictures. A recent case report of a 53-year-old woman with EPSBO after hysterectomy revealed a significant improvement of the symptoms encountered at presentation (nausea, vomiting, and constipation) after systemic administration of methylprednisolone (500 mg/day daily for three consecutive days) [6]. In a multicentre retrospective cohort study, Yang et al. [11] demonstrated lower non-elective surgery rates and fewer adverse events with corticosteroid use among patients with malignant small bowel obstruction. From another perspective, intralesional steroid injection (triamcinolone) along with endoscopic balloon dilation achieved complete resolution of colorectal anastomotic strictures in a series of three patients who had been refractory to repeated dilations, with no septic or procedural complications [12]. In a randomized clinical trial, Gong et al. [13] supported the efficacy of short-term dexamethasone in accelerating recovery from EPSBO. However, long-term prednisolone administration was associated with increased risks of infection and complications, as shown in a large retrospective cohort study [7]. Collectively, corticosteroids may play a significant role in selected cases with anastomotic edema or other causes via reducing inflammation and avoiding the need for reoperation [6].

In essence, multiple mechanisms can contribute to the observed effects of corticosteroids in early postoperative small bowel anastomotic strictures. They can reduce edema and vascular permeability by inhibiting phospholipase A2 and interfering with eicosanoid synthesis [14]. Corticosteroids also suppress inflammatory cell activity, including degranulation of mast cells and cytokine release, which would limit mucosal swelling [15]. Furthermore, corticosteroids interfere with fibrotic scar formation by inhibiting fibroblast proliferation and collagen deposition; therefore, intralesional injection of triamcinolone would help in the prevention of anastomotic stricture recurrence [12]. As a consequence, such effects provide a mechanistic rationale for corticosteroid use in early postoperative anastomotic strictures by suppressing inflammation, reducing edema, and preventing fibrosis.

Despite the above-mentioned benefits, corticosteroids should be used with caution in distinct subsets of patients. An early experimental investigation on rats showed that prolonged or high-dose therapy may impair anastomotic healing via reducing collagen deposition and inhibiting bursting strength [16]. Moreover, clinical data showed that chronic corticosteroid administration may be associated with higher postoperative morbidity and infectious complications despite the comparable rates of leakage and mortality between those who received corticosteroids and those who did not [7]. Furthermore, patients with specific comorbid conditions, such as uncontrolled diabetes, impaired immune responses, and osteoporosis, may be subject to exacerbation of their conditions due to corticosteroids [17,18]. Therefore, their use should be limited to selected patients in whom the potential benefits of anastomotic edema would outweigh the drawbacks.

Importantly, it is crucial to rule out other mechanical causes of obstruction that may require surgery. Accordingly, CT scanning with both intravenous and oral contrast is an invaluable approach in this setting, since it improves diagnostic accuracy and enables accurate interpretation of mechanical obstruction. Furthermore, oral contrast in CT imaging would allow better characterization of luminal patency (partial or complete obstruction). This is because corticosteroid use is more effective in partial obstruction with peri-anastomotic edema, whereas cases with complete obstruction would be more eligible for surgical intervention [19]. Oral contrast would also enhance the ability to exclude anastomotic leak, which is a significant contraindication for steroid therapy, and it necessitates surgical intervention [20]. Therefore, before corticosteroid therapy initiation in early postoperative small bowel anastomotic strictures, we recommend performing CT with oral contrast in all suspected cases to rule out the possibility of leakage and identify those with partial obstruction who are more likely to respond.

## Conclusions

In conclusion, we reported a successful case of using short-course systemic corticosteroids for the management of EPSBO secondary to presumed anastomotic edema. This ultimately led to avoiding the need for reoperation. Supportive evidence showed that corticosteroids are effective in reducing edema and inflammation, as well as improving luminal patency. Accordingly, corticosteroids may be used as an adjunct to conservative management in carefully selected patients with EPSBO due to anastomotic edema rather than complete mechanical disruption. Corticosteroid interventions should ideally be preceded by CT with oral contrast to exclude leaks and to distinguish between complete and partial obstruction. Based on our findings, outcomes appear more favorable in cases of partial obstruction. Future prospective studies with larger cohorts are warranted to better define the safety profile, optimal dosing, and duration of corticosteroid therapy in this specific postoperative setting.
